# Stigma, beliefs and perceptions regarding prostate cancer among Black and Latino men and women

**DOI:** 10.1186/s12889-021-10793-x

**Published:** 2021-04-20

**Authors:** Neha Vapiwala, David Miller, Brenda Laventure, Kristina Woodhouse, Sheila Kelly, Jade Avelis, Cordelia Baffic, Rodney Goldston, Karen Glanz

**Affiliations:** 1 Department of Radiation Oncology, University of Pennsylvania, 3400 Civic Center Boulevard, TRC 4 West, Philadelphia, PA 19104 USA; 2 Department of Radiation Oncology, MD Anderson Cancer Center, Houston, TX USA; 3 Department of Biostatistics, Epidemiology and Informatics, University of Pennsylvania, Philadelphia, USA; 4 JugHead Media, LLC, Philadelphia, PA USA

**Keywords:** prostate cancer, stigma, beliefs, misperceptions, Black, African-American, Hispanic, Latino

## Abstract

**Background:**

Health disparities in prostate cancer (PC) are thought to reflect the complex interplay of socioeconomics, environment and biology. The potential impact of beliefs and perceptions about PC among Black and Latino populations on clinical disparities are not well understood. This qualitative study was conducted to assess current prevalent and pervasive stigma, beliefs and perceptions regarding PC among Blacks and Latinos living in a large metropolitan area, thereby identifying potentially modifiable barriers to care.

**Methods:**

Qualitative data were collected through four separate focus groups of self-identified Black and Latino adult men and women living in Philadelphia to better understand their perceptions of PC diagnosis, screening and treatment. Each focus group was single-sex and conducted by racial/ethnic group in order to assess possible differences in beliefs about PC based on gender and racial/ethnic affiliation. Audio recordings were transcribed verbatim by trained research assistants and qualitative data analysis was conducted using modified grounded theory.

**Results:**

There were a total of 34 participants: 19 Hispanics/Latinos and 15 Blacks, with equal numbers of men and women (n=17). Median age was 57 years (range: 18 to 85 years). Dominant themes that emerged with respect to PC diagnosis included the stigma surrounding this condition and the perceived role of an “unhealthy lifestyle” and certain sexual behaviors as risk factors for PC development. While the majority of participants acknowledged the importance of PC screening and early detection, discussion centered around the barriers to both the interest in seeking medical care and the likelihood of securing it. These barriers included misunderstanding of PC etiology, distrust of the medical profession, and financial/access limitations. Men expressed substantial confusion about PC screening guidelines. In the Black female group, the role of faith and religion in the course of disease was a major theme. Both Black and Latina females discussed the role of fear and avoidance around PC screening and treatment, as well as the prevalence of misinformation about PC in their familial and social communities.

**Conclusion:**

Black and Latino focus groups revealed the existence of cultural beliefs, misunderstandings and fears pertaining to PC which could influence health-related behaviors. Some themes were common across groups; others suggested racial and gender predilections. Future targeted efforts focused on directly addressing prevalent misperceptions among underserved communities in urban settings could help to improve health literacy and equity in PC outcomes in these populations.

**Supplementary Information:**

The online version contains supplementary material available at 10.1186/s12889-021-10793-x.

## Background

Prostate cancer (PC) is the most prevalent non-skin malignancy and the third most common cause of cancer death in American men, with roughly one in ten men diagnosed with PC during their lifetime [1]. Parsing these statistics by race, Black men are 70% more likely to be diagnosed with and over twice as likely to die from PC than White men [2]. It has long been established that Black men are also more likely to develop cancer at a younger age and to present with high-grade, high-risk prostate adenocarcinoma [3]. Although this increased PC incidence and aggressiveness has not been documented in Latino men, PC is still the most commonly diagnosed male cancer in the Latino population [2, 4]. Latinos are in turn recognized as the largest and most rapidly growing minority group in the United States (US) [5]. Although a hereditary predisposition has been suggested by the identification of several genetic variants in Black and Hispanic men that confer an increased risk of PC incidence, the clinical utility of these markers remains under investigation given their absence in the majority of PC patients [6–9].

While research to date has not clearly elucidated the biological mechanisms that underlie the disparate PC incidence and mortality rates observed in various subgroups, studies have consistently implicated the intricate contributory role of socioeconomic factors. Education, occupation, and income clearly impact one’s health status and health care outcomes [10]. For example, members of ethnic minority groups in the US are the least likely to have insurance coverage [11], often due to un- or underemployment. Almost 15% of Black and more than 20% of Hispanic adults in the US are uninsured, compared to less than 10% of those who are non-Hispanic White [12]. Limited access to healthcare contributes to lower rates of PC screening, more advanced stage of disease at diagnosis, and ultimately higher mortality rates [11]. However, even with provision of better healthcare access, Blacks and Latinos, relative to Whites, face more challenging circumstances regarding social determinants of health, such as income and residence location [13]. Furthermore, for those men who are diagnosed and seeking consultation on treatment options, lower educational levels are associated with decreased use of strategies such as shared decision making, which can help patients better understand of their treatment options [14].

Delving into these issues further, there is extensive evidence documenting broad mistrust, fear, and generally negative opinions of healthcare providers among Blacks and Latinos [11, 15–20]; lower rates of screening practices among minority populations relative to Whites are thus hardly surprising [21]. Additionally, minority group members are more likely than non-minority members to seek informal health advice from family members, friends, community centers, and churches, and often do not feel comfortable speaking with healthcare providers from different racial or ethnic groups [18, 19, 22]. Feelings of fear and shame associated with a PC diagnosis further compound the issue of open communication with healthcare professionals, especially when a cancer that can affect physiological (bowel, bladder) and sexual function is involved [23]. When patients are uncomfortable talking about causes, symptoms, and side effects of PC, misinformation, misperceptions, and myths may be perpetuated. In light of the above, researchers have long voiced the need for culturally-sensitive and diverse interventions to address these myths and underlying barriers, and to ultimately increase health-seeking behavior among these at-risk minority groups [24]. Although a number of studies have explored Black men’s views about PC, there are few reports about Latino men’s perceptions, as well as what women of both Black and Latina backgrounds know and believe about PC. Prior work exploring health-seeking behaviors among African American men notes the social influence of female partners [25] and Latino healthcare models emphasize the role of women as “primary forces” in the family, together with tendency of familial involvement in health-related issues [26]. This study aimed to better characterize stigma, beliefs, and perceptions pertaining to PC among Black and Latino men and women residing in an urban community, thereby identifying potentially modifiable barriers to care.

## Methods

### Sample

Eligible participants were adult males and females aged 18 years or older who attended a large and diverse Center City Philadelphia church with predominantly Latino and Black membership. Inclusion criteria included identifying as Latino or Black, and not working in the medical field. People whose primary language was either English or Spanish were included. Women, even those without male partners, were eligible since their opinions are relevant to understanding the cultural beliefs and stigmas in the study population as a whole. Study staff recruited participants via advertising flyers and word-of-mouth, with permission from the church leaders. Two members of the research team have previously attended the church and assisted in obtaining the church leaders’ permission to both recruit for and conduct the focus groups onsite.

### Procedures

Given that this is a pilot study, we sought to have four groups to gather initial data on opinions, but not to reach saturation [27]. Separate focus groups sessions were held for males and females, and for Blacks and Latinos, for a total of four sessions. Each group was peer-led (e.g., a Black man led the group of Black men), given the sensitive nature of the topic and to help the participants feel open to sharing freely [28, 29]. Each focus group was held on a Sunday afternoon in the church building following church services. Two trained research team members (moderator and a note-taker) conducted the focus group sessions. For the Hispanic/Latino groups, a third team member (translator) was available to assist with any language difficulties that might arise.

All moderators participated in an intensive two-day workshop focusing on effective communication skills and led by an experienced qualitative research team. For example, moderators learned how to handle dominant respondents as well as how to ask questions that would elicit the best quality responses without introducing bias. During focus groups, the moderator reminded participants that the sessions were voluntary, confidential, and would be audio recorded. The participants completed and provided written informed consent before the group discussion began and each focus group session lasted approximately 90 minutes. Lunch was provided to participants. The study procedures were approved by the University of Pennsylvania Institutional Review Board.

### Focus Group Survey

All participants completed a brief survey about demographic characteristics and personal and immediate family history of cancer.

### Focus Group Discussion Guide (Supplementary File 1)

A four-part, semi-structured focus group guide was created to help direct the discussion. The first section included an explanation of the research and ground rules for the group. The second part asked general questions about health, such as current health practices, where respondents receive information about health, and when they visit healthcare professionals, while the third section focused more specifically on PC. Participants were then given forms with a series of statements about PC (e.g., “prostate cancer only affects elderly men”) and asked to individually decide whether or not they endorsed these statements. Confronting these statements allowed the participants to reflect on their opinions and this helped bring to mind what the participants thought about these issues. The focus group leaders then led the respondents through discussion on each of the topics. At the end of the group, participants were invited to ask questions about PC and share what they felt were the most important topics discussed during the group. This final, closing segment of the group, after initial discussion had taken place, was primarily for participants to ask questions and did not provide the data analyzed below.

### Analysis Methods

Focus group audio recordings were transcribed verbatim by trained research

assistants. Data analysis was conducted using modified grounded theory [30], in which thematic codes were developed both a priori from study research questions and the focus group guide (Table 1) and de novo from the focus group transcripts (Table 2).

**Table 1:** Endorsed Beliefs

		Latino men	Latino women	Black men	Black women
-Risk factors	-**All Groups**	-General lifestyle Age (disagreement) -Environment Race/ethnicity	-Sexual activity -Race/ethnicity	-Diet -Stress -Blood pressure -Race/ethnicity	-Diet -Sexual activity -Race/ethnicity
-Screening	-Lifestyle factors	-Whether PSA and DRE test the same things		-Whether/how screening can prevent prostate cancer -What age to begin screening -Whether prostate cancer has symptoms -PSAs are unreliable (false positives)	
-Treatment				-Side effects of different types of treatment available -Curability	-Curability

**Table 2:** Other Major Themes

	All Groups	Latino men	Latino women	Black men	Black women
-Screening	-Importance of screening -Importance of knowing family history	-Distinguished between DRE and PSA		-Distinguished between DRE and PSA -Increased awareness of prostate cancer -Role of disparities	-Distinguished between DRE and PSA -Increased awareness of prostate cancer -Role of disparities
-Healthcare seeking			-Financial barriers Women as facilitators -Providers as facilitators -Gender differences in healthcare seeking	-Financial barriers -Women as facilitators	-Financial barriers -Women as facilitators -Gender differences in healthcare seeking
-Communication with providers			-Men more comfortable with male providers	-Providers have financial motivations -Historical cultural distrust of medical profession -Male reluctance to speak to any providers about prostate cancer	-Men more comfortable with male
-Role of religion		-Importance of faith and religion			providers
-Role of stigma	-Prostate cancer is a stigmatized disease		-Threat to sexual identity	-Threat to sexual identity	-Importance of faith and religion
-Fear and avoidance			-Fear of exam process	-Fear of exam process -Fear of positive result -Male avoidance of problem -Male avoidance of telling families	-Threat to sexual identity
-Pushback against stigma /secrecy			-Awareness of misinformation	-Awareness of misinformation -More willing to discuss with age -More willing to discuss after personal experience	-Cultural norm of secrecy around medical problems

A team of three study team members trained in qualitative analysis coded each transcript using NVivo11 [31] and met regularly to review emerging themes, ensure inter-rater reliability, and establish consensus on any discrepancies through coding comparison queries. Final coding comparison queries of the four transcripts showed no major discrepancies between coders. Codes were systematically ranked and used to develop key themes. Two study team members examined relationships among and across codes to develop theories about emerging themes. The team recorded background survey data in a REDCap database and performed descriptive analyses.

## Results

### Focus Group Participant Characteristics

There were a total of 34 participants. The four groups ranged in size from 7-10 participants. There were a total of 19 Hispanics/Latinos and 15 Blacks and equal numbers of men and women (n=17), subdivided as follows: nine Latino men, 10 Latina women, eight Black men, and seven Black women.

Ages ranged from 18 to 85 years, with a median age for all participants of 57 years and a mean of 52 years. This was relatively consistent across the groups, with a slightly higher median among Black women (median age=64 years, mean=62.57, SD= 13.58) and a slightly younger median among Latino men (median age=52, mean =43.33, SD=19.65).

Almost all participants (91.2%) identified as heterosexual. For marital status, 44.1% (n=15) were married, 38.2% (n=13) were single, and 11.8% (n=4) were separated or divorced.

Members’ levels of educational attainment varied. Six (17.6%) of the respondents had less than a high school degree, eight (23.5%) had a high school degree or equivalent, eight (23.5%) has some college or an associate’s degree, six (17.6%) had a bachelor’s degree, and six (17.6%) had a graduate degree. The Black women’s group reported the highest levels of educational attainment, with all members having received at least some college education.

There was also a diverse report of working status. Under half of the respondents were working full-time (n=14, 41.2%), three (8.8%) were working part-time, six (17.6%) were unemployed, eight (23.5%) were retired, and three (8.8%) were disabled or unable to work.

Household income spanned from less than $20,000 to more than $100,000. While four participants did not share their incomes, ten (29.4%) made less than $20,000, nine (26.2%) made $20,000-$49,999, and 11 (32.4%) made more than $50,000. Black women reported the highest household incomes, with 71.4% of participants reporting a total yearly household income of over $50,000 a year.

Participants were asked about their personal history of cancer, the history of cancer in any immediate blood relatives, whether they smoke, and, for men, whether they had ever been screened for PC. No participants reported a personal history of PC; the only types of cancer that participants had personal histories of were breast, colorectal, thyroid, and “other.” A history of cancer in immediate blood relatives was reported by multiple members in every group, and every group had at least one member reporting a family history of PC. The only other cancer family history commonly reported across all groups was colorectal cancer. Among men, Latino men were evenly split between having been screened for PC and not having been screened, while far more Black men had been screened for PC (n=6) than not (n=2).

### Endorsed Beliefs (Table 1)

#### Beliefs about Risk Factors

All groups endorsed lifestyle as a risk factor for PC. Black men and women mentioned diet as a possible cause. Women (both Latina and Black) suggested that sexual activity was related to PC, with one Black woman speaking of masturbation and one Latina woman believing that PC is sexually transmissible.

While there were members of all groups who endorsed non-white race and ethnicity as a risk factor for PC development, there were differing views on the reasons why Blacks and Latinos have higher rates of PC. Black men discussed the role of differences in lifestyle factors between Whites and people of color, specifically citing reasons such as Blacks are more stressed, exercise less often or differently, and tend to have higher blood pressure due to dietary salt intake and/or genetic predispositions. Black women also discussed the role of diet in increasing or decreasing risk of PC, but did not link this to a difference in PC outcomes between racial and ethnic groups. Latino men see lifestyle factors as important in affecting the likelihood of getting cancer; some asserted that it matters more than race or ethnicity. Latina women mentioned a good diet and exercise as ways to keep healthy in general but do not link the lifestyle factors to PC specifically.

#### Beliefs about Screening and Treatment

Members of all four groups endorsed the importance of screening for PC and its role in prevention. Black men, Black women, and Latino males had heard of both the prostate specific antigen (PSA) and digital rectal exam (DRE).

Nonetheless, both groups of men expressed incorrect facts regarding PC screening. Screening tests themselves were discussed, with Black men endorsing mistrust of PSA tests and a belief that they are unreliable, suggesting that there is a high likelihood of false positives. Black men were also unsure about screening recommendations (specifically, at what age to begin screening) and whether screening can actually prevent PC rather than just detect it. Latino men were uncertain about whether the DRE is a screening test for PC or just a test for assessing the size of the prostate.

Both groups of Black participants endorsed the existence of cancer disparities in screening between Whites and minorities, such as this Black male participant who shared, “It was my understanding that Black and Latinos were more likely to die of PC because it goes undiagnosed, not that they get it more, but they die from it because it goes undiagnosed- because they wait until later [to be screened].” Black men endorsed a belief that white men get screened more frequently and therefore can catch and treat PC earlier than non-white men.

Both of the Black groups endorsed misperceptions related to PC treatment and they disagreed about its curability. Specifically, Black men were unsure about what types of treatments are available and what side effects are associated with these treatments.

### Other Major Themes (Table 2)

#### Cultural and Financial Barriers to Healthcare Seeking Behaviors

Black women and men both spoke of cultural differences in healthcare-seeking behaviors. Specifically, Black men discussed historical mistreatment by medical professionals both as something they have experienced personally and as something that has happened to the Black community at large. One man shared that he “came up in a society where black people were treated differently in the hospitals. Much differently.” These early experiences continue to have an effect on how men perceive and interact with members of the medical profession.

Cost is another issue that the focus group members mentioned. Black men and women and Latina women identified finances as a major barrier to healthcare seeking, particularly insurance coverage. Black women shared that providers can be a source of assistance around this issue. One woman stated, “God forbid you did an MRI or CAT scan or something like that, the insurance company won’t pay for that … unless you got a doctor that really … fights for you, you know?”

Given that PC only affects men, gender and heterosexual relationship dynamics also came up in discussion of the disease. Black and Latina women both spoke of men avoiding medical appointments unless they have somebody who “makes them.” Both groups of women identified fear and unwillingness to be seen as weak as driving factors behind male avoidance of healthcare. They also spoke of the important role of women pushing male partners to receive care, which one Black man confirmed by stating, “My wife got me going to the doctor once a year.”

Latina women spoke about gender norms about communicating with peers and suggested that medical issues were unlikely to come up in casual conversation. One woman explained, “Men don’t talk about prostate amongst themselves, all they talk about is about football.” One Black men echoed this idea: “Men in general, they don’t talk about stuff like women talk about stuff … We’re all social beings, but certain things just don’t come up because we just, you know, talk about football or talk about basketball.”

#### Provider-Patient Communication

Some participants identified insurance and the larger medical system as constraints on doctors’ ability to provide the best care. One respondent argued that while doctors want to help their patients, “the system that they’re under today, managed care and everything else; it’s complex... I think there are things they would like to do but perhaps can’t do.”

However, these voices that give doctors the benefit of the doubt are the minority, with suspicion and mistrust as much more common themes. Distrust of the medical profession because of historical wrongs was discussed extensively by Black men, with one participant explaining that because of historical mistreatment of Black Americans by doctors, “that skepticism will remain with us.” Personal experience appears to be a deciding factor in whether men will trust a doctor after “walking in skeptical.” Black men also spoke of a belief that doctors are motivated in decision-making financially by receiving “kickbacks” by issuing specific prescriptions and needing to “charge a lot of money” because of debt from medical school.

In addition to trust, women posited that a doctor’s gender is important for open patient-provider communication. Both groups of women believed that men would feel more comfortable talking to male providers than female providers. While many men also said they would be more comfortable speaking with a male provider, others spoke of the irrelevance of gender in the face of a medical problem. One Latino man shared, “I was in the military for a long time so I couldn’t pick and choose who my doctor was and there were a lot of women doctors and … I didn’t feel squeamish about telling them. I mean if I had a problem, I told them. Like [name] said, I got to get it out.”

#### The Role of Religion

The role of faith and religion in the course of a disease was discussed extensively in the Black female group. Whether disease and death is “God’s will” was not contested, but members disagreed on how much that stance should moderate healthcare seeking.

The importance of having faith (“Rely on the word of God and his promises for us”) and trust in God’s healing power (“God and his mercy healed me”) were endorsed. One Black participant captured the intersection of faith and medicine with a personal story, illustrating that faith and conventional medicine are sometimes tied closely together. She shared, "My dad, grown man of faith, knew something was wrong. Thought he was bleeding hemorrhoids but and he waited and he prayed and prayed. He was asking God to heal him because he wanted to [be] faith healed. And every time he got his answer, it was like, ‘Go to the doctor, go to the doctor,’ but he didn’t go because he said he figured he can worry God into healing him” While religious faith was important to many of the participants, it did not necessarily supersede seeing a doctor or seeking other medical advice.

#### Stigmatization of Prostate Cancer

While all four groups articulated the importance of knowing one’s family history, specifically of cancer diagnoses, Black women discussed a cultural reticence to discuss medical problems within the family (“They just didn’t discuss what they died of, you know, especially in the Black/African-American culture, probably. They didn’t talk about things like that”), particularly if it was a “personal, sexual type situation.” This secrecy was reported to exist not just in men but also in women.

There was consensus among participants that PC in particular is a stigmatized disease among men. Women in both groups and Black men viewed the stigmatization was viewed to come from two sources: the mental and physical discomfort with screening procedures (specifically, the DRE) and because of the prostate’s connection to erectile function. If a man has PC, treatment might mean that his sex life is negatively impacted, which is often not the case for the treatment of other cancers or diseases. One Black man relayed that, “I heard it in another church, they were saying ‘don’t let them take your prostate out, you won’t be able to’ … you know.” Sometimes the link between the prostate and sexual functioning means men are unwilling to talk about the disease at all in order to maintain their image of masculinity: “Everyone wants to be Mr. Man. I’m Mr. Player. Nobody’s gonna talk about nothin’ like that. That’s a whole thing on your manhood” (Black male participant). The potential implications for a man’s sexual experiences makes even discussing PC—not to mention sharing one’s test results or diagnosis—a stigmatized topic among men.

#### Fear and Avoidance

Both Black groups and the Latina group discussed the role of fear and avoidance around PC screening and treatment. Latina women spoke about the fear to see a doctor as a weakness that can also be seen when men are ill. One woman explained, “Men are fearful not like us women. Men are chickens and unlike us strong women who can have children. Men get the flu and they stop working and are shaking.” One participant said that her father-in-law died of PC because he was afraid to go to the doctor.

Latina women also discussed the specific fears surrounding “fear of what they have heard” about the exam process. One woman stated, “[My husband] was afraid of the exam. This is because the men hear other people telling them how the exam is done and they are terrified and they prefer to die with the sickness than to get the exam.”

In contrast to these assertions that men are afraid of the doctor or tests, Black men spoke of fear about the exam’s results, specifically “a fear of finding out that it’s positive,” which was a barrier to even being screened or tested. They also described an unwillingness to discuss results with families if they find out they have PC, with one male subject stating that “they kind of keep it a secret and somehow family members begin to find out.” One respondent suggested that this is due to a sense of fatalism unique to Black men in society today: “I think it’s a quiet, secretive type of shyness, because we reign at everything that’s like, negative, like jail, incarceration, drop-out rates, education, alcoholism. So people kind of give up hope in a sense. They say, ‘Well, hey, something’s gonna kill me.’” The idea is that they as Black men are likely to be the recipients of a negative outcome in society regardless of what they do anyway makes these men resigned and unwilling to share medical news with friends and family. Black women also mentioned men’s opposition to disclosing results, attributing it to an “unwritten kind of thought that if you don’t talk about it, you don’t have to deal with it.” Avoiding talking about a diagnosis is more comfortable for many men than facing the reality of a life with cancer.

#### Pushback Against Secrecy and Stigma

Latina women and Black men both expressed awareness of the existence of misinformation surrounding PC. A Latina woman elaborated on men’s unwillingness to talk to peers about PC, saying that when they do talk about it, “it is only to spread bad information and to spread fear.” A Black man also indicated that peers provide what he described as mixed information, saying, “ … the doctors are saying one thing, because they want to route you a certain way. Church folks who know … or [who] may not have accurate information are saying … this and that, and I think people are listening to people who had different diagnoses and different treatments. They just blurting out different stuff, because it sounds accurate … I’m not going to say misinformation, there’s mixed information … . Person over here had one specific thing done and then he’s telling his friends because it pertains to him, and that’s another specific case. So people are talking but … it’s different stuff.”

Black men reported feeling more willing to speak to one another and provide support either after experiencing a medical problem personally or as they have grown older. One participant reflected on the difference in candor between his current age and his youth, saying, “Back in my twenties, listening to old people talk about that stuff, I had to leave the room. I didn’t want to hear it. And now I find myself being exactly what grossed me out.” Another participant agreed that “most men are uncomfortable talking to each other about it” but said that now that he received treatment for a disease, “I’m not uncomfortable at all; I’ll talk to everybody.”

One Black man denounced the patterns of secrecy around PC as “selfish”, attributing this mentality to age and mentioning the importance of being honest with one’s self about facing diagnoses directly, stating, “I think you’ve gotta get beyond yourself when you’re worrying about not wanting to know. That’s one of the things as I got older, I was like, you know, it’s not just about me. I have to think about my family, the people around you … You gotta get beyond the fear of diagnosis because you gotta think about the big picture of just keeping yourself around for other people who depend upon you. So it’s not just about you and what you’re scared of and what you don’t want to get done. You gotta get beyond that.”

## Discussion

Our study aimed to assess current prevalent and pervasive stigma, beliefs and perceptions regarding PC among Blacks and Latinos living in a large metropolitan area. In doing so, we sought to elucidate culturally-based barriers to care that could inform future interventions. We selected PC as an example of a condition that, despite its highly prevalent nature, may be poorly understood by the public. Frequent and potentially conflicting media headlines regarding cancer etiology, screening and treatment play a role in sowing confusion [32]. Exacerbating matters were dramatic swings within the past decade in the US Preventive Services Task Force’s national screening guidelines – first against and then back to supporting use of periodic PSA testing [33]. These guideline shifts led to significant uncertainty among healthcare providers on how best to counsel men and resulted in a notably increased incidence of advanced-stage PC in the US for the first time since the advent of the PSA test. We also selected PC because of the sensitive nature of a genitourinary cancer diagnosis, expecting that certain themes that emerge from our work can be applied to colorectal, cervical, anal, and other stigmatizing cancers. For some Black and Latino communities, we hypothesized that the aforementioned ubiquitous challenges of assimilating accurate information and making evidence-based decisions are further compounded by cultural and social beliefs surrounding PC, which are likely to differ based on background and gender [22, 25], hence the focus groups conducted in our study. The presence of prevailing “urban myths” coupled with lack of open dialogue in one’s familial and social circles can serve to perpetuate the stigma, shame and fear associated with a potential cancer diagnosis [33]. For a condition like PC that can be highly curable if detected early [1], it is particularly critical to understand these cultural beliefs and perceptions to ensure they do not pose avoidable barriers to timely care. Here we focus on three major themes that emerged from our work that can help inform future strategies and tailored interventions.

One factor that has been previously described in non-White patient populations, which we also saw among our sample, is a mistrust of healthcare professionals, Western medicine, and the system at large [18–20]. Even in our study population of city-dwellers surrounded by a high density of public and private hospitals, participants spoke of obstacles to healthcare seeking and access, including cultural distrust of the medical profession by Blacks and inadequate financial resources, specifically insurance coverage. There was a sense that individuals in the medical field could not be trusted to appropriately care for Black patients, which underlies and complicates the communication barrier between many providers and the patients whom they serve but to whom they cannot culturally relate. In a disease like PC, where there are often multiple therapeutic options from early to advanced stages, lack of shared decision making (due to a lack of trust in the clinician by the patient) and clear understanding of the potential side effects can lead to treatment decisional regret [34]. Unfortunately, most of the published information available for decision making is based on clinical trials in which Black and Latino men are largely underrepresented [35, 36]. Decisions on PC screening, diagnosis, and treatment are thus typically made for the general population based on what we know from research performed in white males, and may omit the important role that trust plays in decision making [35, 36]. This can perpetuate the complicated cycle of mistrust of scientific research and the healthcare system, which in turn contributes to under enrollment of minorities in clinical trials [37, 38]. First steps in addressing some of these issues could include development of culturally-appropriate materials for various minority groups and addressing the perpetual concern of equitable access to and service by healthcare providers.

Another dominant theme that emerged was the influence of beliefs about gender roles, stereotypes and sexuality on perceptions of PC and healthcare seeking. Women from both racial/ethnic groups specifically (and incorrectly) endorsed male sexual activity as a risk factor for PC development. Women also believed that men do not seek healthcare unless made by another person (often a wife) because of fear, pressure to be the primary “breadwinners”, or an unwillingness to be seen as ill or weak, and yet neither male group endorsed this view. Interestingly, Latina women shared key perceptions with both the Black men and women regarding with respect to the stigmatized nature of PC; all three groups spoke of men’s perception of PC as a threat to sexual identity and masculinity (machismo), rooted in fear of a detrimental effect on sexual performance. Acknowledgment of this concern regarding sexual identity and performance by Black men but not the Latino men suggests a particular reticence in the latter population that could be a function of the group setting, potentially addressable through one-on-one conversations with trusted male peers, mentors or healthcare professionals. Relatedly, participants did differ along gender lines with respect to perceptions on differences in willingness to speak to others about health problems, specifically that women are more communicative and men are unwilling to talk to each other. Of note, women believed men would be more comfortable speaking to a male provider about PC, as opposed to a female provider, although this was not unanimously endorsed by male participants. Participants from all groups did note that secrecy and stigmatization can stifle open conversations around PC. These findings underscore how the fears of morbidity and mortality that naturally arise with a cancer diagnosis can be magnified in communities that value privacy and a culturally-engrained reticence to discuss medical problems in general. These fears, along with the intertwined relationship among prostate health, sexual functioning, and ideas about gender roles, suggest that PC is a sensitive topic closely tied to perceptions of lost masculinity and virility. Public health messaging to educate Black and Latino communities about stigmatized cancers such as PC should continue to take into account these fears in a manner that is honest but reassuring.

A third important theme that emerged from our work pertains to the perception among Blacks and Latinos of the differences in their outcomes and experiences relative to other groups. Both the Black and Latino participants believed their respective race/ethnicity was a risk factor for PC, even though increased PC incidence and aggressiveness has been documented in Black but not Latino men [4]. Participants postulated that one cause was racial disparities in screening, with the assumption that white men receive more screening and treatment. Of particular note, despite our study population of churchgoers, it was the Black women who strongly endorsed faith and religion as a mediator in the progression of a disease, and felt that this influences attitudes towards medical treatment. This also suggests the potential value of a multifaceted and religiously sensitive approach to the dissemination of scientifically accurate information among subgroups with deeply rooted beliefs in the power of healing though religion and spirituality.

Our study has several limitations, namely the small sample size inherent to qualitative studies of this kind and the single primary source of subject recruitment, thus limiting the representativeness of our population [39]. The effect of speaking in a small group may in itself influence the answers, as might the varying personalities of the participants, although the moderators were skilled in ensuring that all individuals had an opportunity to contribute. Desirability bias is another important limitation that may have influenced focus group participants' narratives [39].

Despite these limitations, we believe that understanding the cultural- and education-related barriers to minorities’ engagement and trust of the healthcare system is a key step to reducing disparities and improving outcomes. Identifying the stigmatizing beliefs and perceptions can help to develop barrier reduction strategies rooted in modern, culturally relevant approaches for information dissemination. Our work identified some beliefs and perceptions regarding a highly prevalent but potentially misunderstood and stigmatized cancer that were common among urban Black and Latino men and women, but also some that appeared to be unique to certain subgroups. To further explore the validity of the dominant themes we uncovered, we will next conduct a survey among a much broader sample of Philadelphia-area Black and Latino community members. Specifically, we will explore the depth of existing knowledge about PC and its risk factors and ascertain barriers to routine screening or to seeking attention in the event of symptoms. Integral to this will be exploration of the role of gender roles, sexuality, and religion, as well as the impact of attitudes towards providers and the medical profession on knowledge regarding stigmatized cancers such as PC. Combining our survey results with these important qualitative findings described here will guide the future creation of tailored educational materials. These materials will attempt to help fill knowledge gaps and dispel misconceptions, focusing on reduction of barriers to appropriate utilization of health services.

## Conclusion

Black and Latino focus group participants revealed the existence of cultural beliefs, misunderstandings and fears pertaining to PC which could influence health-related behaviors. Some themes, such as mistrust of the healthcare system, the intersection of PC with gender-based roles and sexuality, and reasons underlying differing cancer outcomes, were common across groups. Other themes suggested racial and gender predilections. Future targeted efforts, focused on directly addressing prevalent misperceptions as potentially modifiable barriers to care in underserved urban communities, could help to improve health literacy and in turn equity in PC outcomes in these populations.

## Supplementary Information

**Supplementary File 1.**

## Data Availability

The datasets used and/or analyzed during the current study are available from the corresponding author on reasonable request.

## Abbreviations

PC: Prostate cancer
DRE: Digital rectal exam
