# Supplementary material for: Stigma, beliefs and perceptions regarding prostate cancer among Black and Latino men and women
Source: BMC Public Health. 2021 Apr 20;21:758. doi: 10.1186/s12889-021-10793-x (PMC8056613; doi:10.1186/s12889-021-10793-x)
Supplement: Supplementary file 1 [file 12889_2021_10793_MOESM1_ESM.docx]

# Appendix 1

**Prostate Cancer Myths and Misperceptions**

**Focus Group Discussion Guide**

| **I. Introduction** |  |  |  |  |  |  |  | **5 minutes** |
| --- | --- | --- | --- | --- | --- | --- | --- | --- |

1. **Introductions**
   1. Introduce yourself
   2. Introduce team members – Names and roles today (note taker, observer)
   3. Ask participants to go around table and introduce themselves – First name, a favorite hobby, and why they were interested in coming today
2. **Research explanation**
   1. You’re here today to help me learn more about your thoughts on health and prostate cancer.
   2. Everyone has a packet of paper and pen in front of them. We will use these later in the session. I will provide further instructions at that time.

**4. Ground rules**

Before we start, I have a few guidelines or “ground rules” to help the group run smoothly:

1. There is no right or wrong answer to these questions – we want you to be honest and to respect others’ opinions, even if you disagree with them. Everyone will have a chance to speak.
2. Only one person should speak at a time. As you know we are recording the focus group, and the audio recorder can only pick up one voice at a time.
3. I may call on you to share with the group because we want to make sure we hear from everyone; however, your participation is voluntary. You do not have to answer any questions you don’t want to.
4. If needed, I may interrupt at times to keep the discussion moving forward and on-topic.
5. Before we begin, please make sure that cell phones and anything else that might make noise are turned off and put away.

Is everyone ready? Let’s begin.

| **II. Health** |  |  |  |  |  |  |  |  | **15 minutes** |
| --- | --- | --- | --- | --- | --- | --- | --- | --- | --- |

**First, let’s talk about health in general.**

1. **What do you do to keep yourself healthy?** *(3-4 min.)*
   1. *For female groups: Probe for how they contribute to keeping the men in their lives healthy.*
2. **Where do you go for information about your health?** *(3-4 min.)*
   1. *Probe for similarities and differences as to where they get general health information.*
   2. *If participants do not seek health information, probe to explore that too.*
   3. *For female groups: Probe for where they go for information about men’s health/how to keep the men in their lives healthy.*
3. **Where do you go for information about cancer, specifically?** *(3-4 min.)*
   1. *Probe for similarities and differences when seeking general health information versus cancer information.*
   2. *Where do you go for information about prostate cancer?*
   3. *If participants do not seek cancer information, probe to explore that too.*
4. **When do you go to the doctor?** *(3-4 min.)*
   1. *If participants do not go to the doctor, probe to explore that too.*
   2. *For female groups: When do the men in your life go to the doctor? Describe your role in that decision/process.*

| **III. Myths and Misperceptions** |  |  |  |  |  | **50 minutes** |
| --- | --- | --- | --- | --- | --- | --- |

1. **Instructions**
   1. Everyone has a packet of paper in front of you. Each sheet of paper is about a topic related to prostate cancer, and there are several statements about each topic. For each topic, I will first ask you to write down whether you agree or disagree with each statement, and then we will discuss the statements as a group.
   2. Keep in mind that my job during today’s focus group is to learn information from YOU, and not to be a teacher or educator. However, I realize that you may have questions about the prostate cancer topics that we discuss as a group. I will leave a few minutes at the END of today’s session to address specific questions, including how a physician would answer those questions.
2. **Discussion of prostate cancer topics and accompanying statements** *(~12 min.*

*per topic)*

- 1. The first topic is prostate cancer risk. Please read each statement, and fill in the checkbox to indicate whether you agree or disagree with each statement about prostate cancer risk.

*(Participants fill out sheet for current topic)*

- 1. Now that you’ve had a chance to agree or disagree with the statements, let’s talk about your thoughts about each statement.
  2. *Read first statement out loud.* By a show of hands, how many of you agree with this? How many of you disagree? *(Note taker will help count and record numbers).*
  3. *Discuss participants’ reactions to the current statement.*

Sample Probes: *Why do you agree?* *Why do you disagree?* *How did you learn about…?* *Tell me more about…*

- 1. Repeat steps c-d for each statement within the current topic.

1. **Repeat Step 2 for each topic.** Topics include:
   1. Prostate Cancer Risk
   2. Prostate Cancer Screening
   3. Prostate Cancer Symptoms and Treatment
   4. Prostate Cancer Outcomes

| **IV. Debrief and Wrap Up** |  |  |  |  |  | **15 minutes** |
| --- | --- | --- | --- | --- | --- | --- |

1. **Educational debrief** *(5-10 min.)*
   1. Now, we’ll take a few minutes to answer any questions you have about the topics related to prostate cancer that we just discussed.

*Dr. Vapiwala has prepared notes that offer a physician perspective on the topics addressed. Use these notes as a tool to answer questions. If there is something we are not comfortable answering, ask if we can follow up with participant(s) after the session with further information.*

1. **Important topics/statements** *(5-10min.)*
   1. Our team will be developing educational materials (like brochures or flyers) to share information and address myths about prostate cancer. In your opinion, which topics/statements that we discussed today are the most important to include?
2. **Wrap up** *(1 min.)*
   1. Are there any additional questions or last thoughts before we wrap up?
   2. *Thank participants for their time.*
   3. *Distribute participant incentives.*

# 1.PROSTATE CANCER RISK

**Directions :** Read each statement, and fill in the checkbox

to indicate whether you *agree* or *disagree* with the statement. Write

down any comments about each statement.

| **STATEMENT** | **AGREE** | **DISAGREE** |
| --- | --- | --- |
| Prostate cancer only affects elderly men. | □ | □ |
| It is important to know if anyone in your immediate family had prostate cancer. | □ | □ |
| Black and Latino men are more likely to get prostate cancer than white men. | □ | □ |

COMMENTS:

_______________________________________

_______________________________________

# 2.TALKING ABOUT PROSTATE CANCER

**Directions :** Read each statement, and fill in the checkbox

to indicate whether you *agree* or *disagree* with the statement.

Write down any comments about each statement.

| **STATEMENT** | **AGREE** | **DISAGREE** |
| --- | --- | --- |
| Prostate cancer is a disease that most people are uncomfortable talking about with healthcare providers. | □ | □ |
| Prostate cancer is a disease that most men are uncomfortable talking about with each other. | □ | □ |
| Prostate cancer is a disease that most men are uncomfortable talking about with women. | □ | □ |

COMMENTS:

_____________________________________

_____________________________________

_____________________________________

# 3.PROSTATE CANCER RISK

# AND SCREENING

**Directions :** Read each statement, and fill in the checkbox

to indicate whether you *agree* or *disagree* with the statement.

Write down any comments about each statement.

| **STATEMENT** | **AGREE** | **DISAGREE** |
| --- | --- | --- |
| If a man gets routine PSA testing *(the prostate cancer screening blood test)*, it reduces his chances of getting prostate cancer. | □ | □ |
| All men should get annual PSA testing. | □ | □ |
| If a man’s PSA test results are not normal, it means that he has prostate cancer. | □ | □ |

COMMENTS:

__________________________________________

# 4.PROSTATE CANCER SYMPTOMS

# AND TREATMENT

**Directions :** Read each statement, and fill in the checkbox

to indicate whether you *agree* or *disagree* with the statement.

Write down any comments about each statement.

| **STATEMENT** | **AGREE** | **DISAGREE** |
| --- | --- | --- |
| If a man doesn’t have symptoms, he doesn’t have prostate cancer. | □ | □ |
| If a man has prostate cancer, he should start treatment right away. | □ | □ |
| Prostate cancer treatment always has negative effects on a man’s sex life. | □ | □ |

COMMENTS:

_____________________________________ _____________________________________

_____________________________________

# 5.PROSTATE CANCER OUTCOMES

**Directions :** Read each statement, and fill in the checkbox

to indicate whether you *agree* or *disagree* with the statement.

Write down any comments about each statement.

| **STATEMENT** | **AGREE** | **DISAGREE** |
| --- | --- | --- |
| If a man gets prostate cancer, he will die from the disease. | □ | □ |
| Prostate cancer can always be cured. | □ | □ |
| When prostate cancer treatment is complete, there are no long-lasting effects from treatment. | □ | □ |

COMMENTS:

_______________________________________
